# Delphi consensus methodology to gauge expert perspectives on smoking prevention, cessation and harm reduction in Italy

**DOI:** 10.3389/fpsyt.2025.1349265

**Published:** 2025-01-31

**Authors:** Pasquale Caponnetto, Vincenzo Contursi, Francesco Fedele, Fabio Lugoboni, Salvatore Novo

**Affiliations:** ^1^ Department of Educational Sciences, Section of Psychology, University of Catania, Catania, Italy; ^2^ Center of Excellence for the Acceleration of Harm Reduction (CoEHAR), University of Catania, Catania, Italy; ^3^ Cardiovascular Area, Italian Interdisciplinary Society for Primary Care, Bari, Italy; ^4^ Department of Cardiovascular, Respiratory, Nephrological and Geriatric Sciences, Istituto Nazionale per le Ricerche Cardiovascolari (INRC), Bologna, Italy; ^5^ Department of Medicine, University Hospital of Verona, Verona, Italy; ^6^ Department PROMISE, University of Palermo, Palermo, Italy

**Keywords:** Delphi consensus, electronic cigarettes, heated tobacco products, Italy, public health, smoke-free products, smoking cessation strategies, tobacco harm reduction

## Abstract

The role of smoke-free alternatives to cigarettes for tobacco harm reduction remains controversial. This study was conducted to understand the perspectives of a panel of Italian experts on this topic. Using Delphi consensus methodology, expert opinions on the use of smoke-free alternatives, tobacco harm reduction and anti-smoking legislation were gathered and analyzed. In July 2022, a Scientific Committee, including five members, proposed 38 statements spanning three areas: (1) harm from tobacco smoking and strategies for harm reduction; (2) smoke-free alternatives to cigarettes; and (3) anti-smoking legislation. Between August and November 2022, the Expert Panel, including members of the Scientific Committee and 15 other key opinion leaders, voted on the statements in two rounds. Consensus was achieved on 24 of 38 statements. The results emphasized the persistent national health threat posed by tobacco smoking in Italy, with a smoking prevalence of 20–24% between 2007 and 2022. Emphasizing harm reduction as a pivotal public healthcare strategy, the Expert Panel agreed on 10 statements related to smoke-free alternatives, but underlined the need for further research despite promising initial findings. The Expert Panel also reached consensus on six statements regarding anti-smoking legislation, stressing the importance of crafting and upholding rigorous anti-smoking laws that are consistent with World Health Organization guidelines. This pioneering Delphi consensus statement illuminates the complicated debate regarding the role of smoke-free alternatives for tobacco harm reduction in Italy. The findings highlight the evolving nature and advocate the need for ongoing discussions and further research on this important issue.

## 1 Introduction

The prevalence of smoking has decreased in some countries during the past 30 years; however, the overall number of smokers worldwide has increased from 0.99 billion in 1990 to 1.14 billion in 2019 due to population growth (1). As a result, tobacco smoking continues to pose a significant health challenge, leading to substantial morbidity and mortality, as well as contributing to negative societal consequences, such as increased healthcare costs (1, 2). In 2019, tobacco smoking was responsible for an estimated 8 million deaths globally and was the leading cause of death and disability among men (1). In Italy, the prevalence of tobacco smoking remains high, at approximately 20.5% in 2023, despite declining from the recent peaks of 24.2% in 2022 and 22.0% in 2019 (3, 4).

The current tobacco control measures fall short and are unlikely to lead to the achievement of the World Health Organization (WHO) objective of a 30% reduction in the prevalence of smoking by 2030 (5). A fundamental change is needed to promote substantial progress in this area.

Tobacco harm reduction is commonly defined as a public health strategy that seeks to prevent or reduce the damage caused by the toxins generated by tobacco combustion for people who wish to continue smoking or who are unable to quit, rather than aiming at complete abstinence from nicotine use (6–8). The use of tobacco results in nicotine addiction and, therefore, harm reduction strategies have the potential to improve outcomes in those who do not give up tobacco smoking (6, 9, 10).

Over the past decade, smoke-free alternatives to tobacco, including electronic cigarettes, heated tobacco devices and orally administered tobacco- or nicotine-based products have become popular, yet controversial substitutes for tobacco cigarettes among smokers worldwide (11–17). Compared with conventional cigarettes, smoke-free alternatives offer a substantial reduction in exposure to toxic chemicals; for this reason, these alternatives can be used to reduce the harm caused by cigarette smoke and as aids for smoking cessation (6, 7, 18–22). One review found that the prevalence of tobacco smoking is lower, particularly among young people, in countries that have higher rates of adoption of smoke-free tobacco alternatives, such as the United Kingdom, Sweden, Norway, New Zealand and Japan, compared with other countries (23).

Despite their growing use, the role of smoke-free alternatives for tobacco harm reduction remains a subject of debate. The scientific and medical communities have addressed the overall potential benefit and harm of these alternatives, with divergent opinions on their efficacy, safety and societal implications (24–28). This controversy underscores the need for consensus on the role of smoke-free alternatives in reducing the burden caused by tobacco smoking. Given this context, the objective of this study was to formulate a consensus on the role of smoke-free products in reducing the harm caused by tobacco.

## 2 Methods

### 2.1 Study design

This study was funded by Philip Morris Italia, a subsidiary of Philip Morris International, a tobacco company that has invested more than $12.5 billion in the development of alternative, less harmful products aimed at reducing the harm caused by tobacco use (https://www.pmi.com/, accessed December 3, 2024). The study employed a Delphi consensus methodology, consisting of two rounds of voting. In July 2022, a virtual meeting of a Scientific Committee that consisted of five specialists in various therapeutic areas (see **Supplementary Methods** for the list of members) was convened to draft a list of statements.

The statements covered three general areas: harm from tobacco smoking and strategies for harm reduction, smoke-free alternatives to cigarettes, and anti-smoking legislation. Each statement expressed an opinion regarding a question of importance in smoking harm reduction, on which there was disagreement or no strong agreement among members of the Scientific Committee. The statements were formulated to be clear and specific (e.g., by avoiding double negatives) to fully express all the dimensions of the problem.

The statements were submitted for online voting to an Expert Panel, which, in addition to members of the Scientific Committee, included 15 key opinion leaders who were members of several Italian scientific societies. Thus, the Expert Panel comprised a total of 20 members (see **Supplementary Methods** for the full list).

To ensure the anonymity of the online voting process, a computer tool was used in which clinicians received a questionnaire via email containing only the questions without indicating the name of the respondent. In no way was it possible to trace the identity of the respondent. This procedure guaranteed that experts could express their genuine opinions without external pressure or influence.

### 2.2 Selection of the Expert Panel

The selection of the Scientific Committee and the key opinion leaders was conducted through the involvement of Italian scientific societies from various disciplines relevant to the project. These societies were invited to participate, and those who accepted the invitation designated either their president or another representative knowledgeable about the topic to act as project contacts. From this pool, the five members of the Scientific Committee were selected based on their extensive experience and recognized expertise on the subject, as evidenced by their scientific publications and initiatives undertaken within the country. Similarly, the 15 key opinion leaders were chosen for their demonstrated expertise and contributions to the field of smoking prevention, cessation, and harm reduction. The Delphi panel was formed with no input from the sponsor. The selection of a diverse group of 20 experts helped ensure a wide range of perspectives, reducing the likelihood of bias.

Working independently, the Scientific Committee ensured a balanced and comprehensive set of statements, focusing on areas where there was disagreement or no strong agreement among members. To ensure integrity and objectivity, all phases of the study, including data collection, analysis, and interpretation, were independently executed by the members of the Expert Panel.

The rigorous nature of the Delphi consensus methodology, combined with additional safeguards, minimizes any potential influence on the panel while ensuring a balanced aggregation of expert opinion.

Members of the Expert Panel were asked to rate each statement on a 9-point Likert scale, in which 1 indicated maximum disagreement and 9 indicated maximum agreement. The 9-point scale was chosen because the odd number of choices would reduce uncertainty while providing the optimum level of precision. Members of the Expert Panel were encouraged to rate all statements. Responses were collected online and were anonymous, to further mitigate potential biases. This anonymity allowed experts to express their genuine opinions without external pressure or influence.

Responses of the members of the Expert Panel were analyzed by an independent methodologist. This independent analysis added an additional layer of objectivity to the process, ensuring that data interpretation was free from sponsor influence.

The level of agreement was categorized as low (Likert scale scores 1–3), moderate (scores 4–6), or high (scores 7–9). Consensus was considered to have been reached on a statement if over 85% of respondents expressed levels of agreement that fell into one of the three categories (low, moderate, or high). These statements would be included in the final list of recommendations. Members of the Expert Panel were considered to have not reached consensus regarding a statement if the proportion of respondents who expressed high or low agreement and the proportion of respondents who expressed moderate agreement collectively exceeded 90%. The Scientific Committee would then discuss and revise such statements before submitting them to the Expert Panel for the second round of voting. If respondents expressed an even wider range of agreement levels on a statement, that statement was considered unlikely to reach consensus and was withdrawn from the subsequent steps. During the first round of voting, members of the Expert Panel could propose additional statements.

The first round of voting took place between August and September 2022, and the second round of voting was in November 2022. At the end of November 2022, the Scientific Committee met to discuss the second round of voting and to prepare the Expert Opinion statement.

In May 2023, the PubMed database was searched for relevant articles (see **Supplementary Methods** for the literature search strategy). All the experts were provided with the scientific literature on the subject to consult before the voting process.

## 3 Results

In total, 38 statements were developed across the three areas of interest: 11 statements in Area 1 (harm from tobacco smoking and strategies for harm reduction), 19 in Area 2 (smoke-free alternatives to cigarettes) and eight in Area 3 (anti-smoking legislation).

During the first round of voting, consensus was reached regarding 17 statements, while members of the Expert Panel did not reach consensus regarding 19 statements (**Supplementary Figure 1**). In addition, opinions diverged on two statements to such an extent that consensus was considered unlikely. These statements were withdrawn from the second round of voting. Following the first round of voting, members of the Scientific Committee discussed and revised the 19 statements on which respondents did not reach consensus.

During the second round of voting, consensus was reached regarding seven additional statements (**Supplementary Figure 1**). Members of the Expert Panel did not reach consensus regarding 12 statements.

The final version of the statements is presented in **Table 1**. In Area 1, consensus was reached for eight statements (Statements 1 and 3–9). In Area 2, consensus was reached on 10 statements (Statements 19–21, 23–26 and 28–30). In Area 3, the panel reached a consensus on six statements (Statements 31–34, 37 and 38).

**Table 1:** Final statements and the outcome of the two-round Delphi process.

No.	Statement	Outcome	
Area 1: Harm from tobacco smoking and strategies for harm reduction			Agreement, %
1	Cigarette smoking represents a national health emergency which must be confronted with high priority throughout our country.	Consensus	100
2	*The increase in deaths due to cigarette smoking and the growth of smoking-related morbidities are principally caused by substances produced during the combustion process.*	Not reached consensus	76.5
3	*The Ministry of Health of Italy should invest more time and resources in programs aimed at helping smokers quit (e.g., antismoking centers, toll-free hotlines, etc.).*	Consensus	94.1
4	The Ministry of Health of Italy must invest time and resources in programs aimed at spreading the awareness and increasing the knowledge of the concept of harm reduction as it relates to smoking.	Consensus	100
5	*The Ministry of Health of Italy should invest time and resources in information programs aimed at increasing the awareness of smoke-free alternatives for adult smokers unable or unwilling to quit smoking.*	Consensus	88.2
6	Public heath can be improved via the implementation of political strategies, programs, services and initiatives with the goal of reducing the effects of damaging behaviors to a minimum, despite the likelihood that such behaviors may not be eliminated.	Consensus	100
7	Reduction of the risks of smoking is a medical strategy, and not of any other nature, on the same level as previous humanitarian approaches adopted successfully by the scientific community in the prevention of certain diseases (e.g., low-salt, low-fat and low-sugar diets, etc.).	Consensus	100
8	*The reduction in the risks of smoking is a medical strategy, equally important as the methods used in the fight against and in the prevention of other chemical dependencies.*	Consensus	88.2
9	Harm reduction is an approach which has the objective of reducing to the lowest possible levels any impacts on health caused by damaging behaviors, even if such behaviors cannot be entirely eliminated.	Consensus	100
10	*Harm reduction can be an adequate approach to confront the problem of cigarette smoking in adult smokers who are unwilling or unable to quit.*	Not reached consensus	76.5
11	*Assisting smokers to transition from typical cigarettes to less-damaging options (such as those that supply nicotine without tar) represents a valid public health strategy (both on a personal and on a public level).*	Not reached consensus	52.9
Area 2: Smoke-free alternatives to cigarettes			
12	*Smoke free alternatives to cigarettes (such as electronic cigarettes, heated tobacco devices or orally administered tobacco- or nicotine-based products) may play a role in the reduction of harm associated with smoking traditional cigarettes to both the individual and to the society.*	Not reached consensus	56.8
13	*The exclusive use of smoke-free alternatives (such as electronic cigarettes, heated tobacco devices or orally administered tobacco- or nicotine-based products) offers a practical and acceptable alternative for adult smokers who would otherwise continue to smoke cigarettes.*	Not reached consensus	76.5
14	*As stated by the FDA, the current scientific evidence regarding the decreased levels of damaging or potentially damaging chemicals produced by electronic cigarettes in comparison with traditional cigarettes is sufficient to consider them products of reduced risk.*	Not reached consensus	70.6
15	*Real-world evidence has revealed that electronic cigarettes are equally effective as the drugs used for smoking cessation* (31).	Not reached consensus	64.7
16	*The dual use of both traditional cigarettes and smoke-free alternatives must be avoided, as it is an ineffective and useless in the long term.*	Not reached consensus	82.4
17	Transitioning from smoking traditional cigarettes to the exclusive use of smoke-free products is equivalent to quitting smoking.	Withdrawn	100
18	For female smokers during pregnancy, transitioning from smoking traditional cigarettes to the exclusive use of smoke-free products is a much safer approach, which may help the woman to completely quit smoking and may to protect her from possible relapse.	Withdrawn	100
19	*Current scientific evidence must be confirmed by sufficiently long-term studies in order to verify whether smoke-free alternatives are associated with a greater reduction in smoking-related pathologies compared with traditional cigarettes.*	Consensus	88.2
20	The cessation of the use of any nicotine- or tobacco-based products remains the gold-standard for which both doctors and patients must aim, reserving the use of alternative products for adult smokers who wish to continue smoking as a harm reduction strategy.	Consensus	100
21	Providing accurate information regarding smoke-free alternatives (such as electronic cigarettes, heated tobacco devices or orally administered tobacco- or nicotine-based products) to adult smokers who would otherwise continue smoking is a step in the right direction for society as a whole and should be regarded as integral to public health policy.	Consensus	100
22	*For adult smokers who would otherwise continue smoking, the government should foster the sharing of scientific information regarding smoke-free products in order to favor informed decision making.*	Not reached consensus	76.5
23	Scientific organizations should use their activities (as well as congresses) in order to promote information and training of healthcare professionals on smoking-related issues and smoking cessation strategies.	Consensus	100
24	Scientific organizations should use their activities (as well as congresses) in order to promote information and training of healthcare professionals regarding alternative options available to adults who decide to not quit smoking.	Consensus	100
25	It would be useful for healthcare professionals to receive additional information on the harm reduction strategies and on the alternative smoke-free products, based on the most up-to date scientific findings available.	Consensus	100
26	It would be useful for healthcare professionals to receive additional information on how other nations have evaluated the risks of smoke-free products relative to traditional cigarettes.	Consensus	100
27	*Contrary to the guidelines suggested by the FDA and the NHS, some public health organizations tend to emphasize the potential risks of smoke-free products, while overlooking their benefits. This approach can have negative repercussions on public health.*	Not reached consensus	70.6
28	There is a lot of disinformation coming from the media regarding the subject of potential risks of smoke-free alternatives.	Consensus	100
29	The scientific evidence on the strategy of tobacco harm reduction and on cigarette alternatives that comes from both independent and industry sources should be discussed and reviewed transparently at appropriate scientific meetings by regulatory authorities based on evidence-based medical principles and without prior preconceptions.	Consensus	100
30	The scientific evidence on the strategy of tobacco harm reduction and on cigarette alternatives that comes from both independent and industry sources should be discussed and reviewed transparently, at appropriate scientific meetings by the medical-scientific community based on evidence-based medical principles and without prior preconceptions.	Consensus	100
Area 3: Anti-smoking legislation			
31	Policies ought to continue to deter people from beginning to smoke any type of product and to encourage them to quit.	Consensus	100
32	An adequate regulatory framework should be used to monitor the undesirable consequences and to reduce as much as possible the initiation of smoking in adolescents and young adults.	Consensus	100
33	Interventions should be proportional to the risk/damage of tobacco and smoke-free alternatives (such as electronic cigarettes, heated tobacco devices and orally administered tobacco- or nicotine-based products).	Consensus	100
34	*Interventions should follow a common sense-based approach: more damaging products, such as cigarettes, should be subjected to more restrictive regulations.*	Consensus	94.1
35	*In Italy, thousands of adults will likely continue to smoke, despite the availability of existing therapies; these people should have the opportunity to transition to less risky alternatives. An adequate regulatory framework should recognize that not all tobacco products have the same risk profile.*	Not reached consensus	76.5
36	*An adequate regulatory framework should offer adult smokers access to less damaging smoke-free products and the necessary information to allow them to make informed decisions regarding their health.*	Not reached consensus	82.4
37	*An adequate regulatory framework should create a way for the rigorous scientific evaluation of novel smoke-free products and their relative health risks.*	Consensus	88.2
38	*An adequate regulatory framework should allow consumers to receive scientifically substantiated information regarding new products with the objective of avoiding misleading claims.*	Consensus	88.2

Statements in italics were modified after the first round of voting.

Agreement indicates the percentage of respondents whose ratings fell into the same agreement category, i.e. low agreement (Likert scale scores 1–3), moderate agreement (scores 4–6), high agreement (scores 7–9).

FDA, The United States Food and Drug Administration; NHS, The National Health Service of Great Britain.

### 3.1 Area 1: harm from tobacco smoking and strategies for harm reduction

Members of the Expert Panel agreed that cigarette smoking is a national health emergency that requires urgent attention. The prevalence of smoking in Italy has remained relatively stable between 2007 and the present (3, 27, 29). Between 2007 and 2016, 20% and 22% of the Italian population were cigarette smokers (27, 29), with the prevalence of smoking increasing from 22% in 2019 to 24% in 2022 (3). As discussed above, the prevalence of smoking appears to have declined in 2023; however, it still remains above 20% (4). These data highlight the gravity of the tobacco smoking problem in Italy.

In addition, the members of the Expert Panel agreed that harm reduction can be a valuable public health strategy for reducing the negative impact of damaging behaviors in individuals who are unable or unwilling to refrain from such behaviors, and that the Ministry of Health of Italy should invest in promoting awareness of harm reduction among smokers, including the use of smoke-free alternatives. The effectiveness of the harm reduction approach has been demonstrated with regard to other damaging behaviors, such as alcohol and drunk driving, as well as drugs and injection-related harm (30).

### 3.2 Area 2: smoke-free alternatives to cigarettes

The Expert Panel concurred that current scientific evidence while promising requires further studies to establish the risk-reduction potential of smoke-free products in comparison to traditional cigarettes. A systematic review of 56 studies, 29 of which were randomized controlled trials, concluded that nicotine-containing electronic cigarettes increased tobacco smoking quit rates compared with nicotine replacement therapy (31). The review also detected no evidence of harm from nicotine-containing electronic cigarettes, a finding that was limited by the relatively short duration of follow-up (31). A subsequent analysis showed that the use of nicotine-containing electronic cigarettes was associated with a reduction in the levels of biomarkers of potential harm, including exhaled carbon monoxide, nitrosamines, polyaromatic hydrocarbon metabolites and other known carcinogens, compared with conventional cigarettes and the combined use of electronic and conventional cigarettes (32).

The benefits of long-term use of smoke-free products versus conventional cigarettes were demonstrated in a study of patients with chronic obstructive pulmonary disease (COPD) who were smokers (33). In the study, objective and subjective outcomes were compared between 20 COPD patients who used electronic cigarettes and 19 age- and sex-matched controls who continued to smoke. After 5 years of follow-up, the self-reported mean number of conventional cigarettes smoked per day was significantly lower in patients who used electronic cigarettes than in those who continued to smoke (1.4 vs 18.3; p<0.001). Furthermore, in the electronic cigarette group, the mean annual COPD exacerbation rate and mean COPD assessment tool (CAT) scores (higher scores indicate greater impact of COPD) were significantly decreased compared with baseline (p<0.001 and p=0.020, respectively), and the mean 6-minute walk distance (6MWD) had significantly increased from baseline (p=0.005). In contrast, for patients who continued to smoke, the changes in these parameters from baseline were not statistically significant (33).

Another study subsequently compared outcomes after 3 years of follow-up in 19 patients with COPD who used heated tobacco products and 19 age- and sex-matched controls who continued to smoke (34). Similar to the previous study, heated tobacco products were associated with a significant reduction from baseline of the mean number of cigarettes smoked per day, annual exacerbation rate, CAT score and 6MWD at 3 years, but these outcomes were not significantly different in patients who continued to smoke (34).

Further supporting the benefit of smoke-free alternatives versus cigarette smoking is the observation that rates of tobacco smoking are lower in countries with higher adoption rates of smoke-free alternatives than in countries with lower adoption rates (23).

However, there are several unanswered questions regarding smoke-free alternatives to tobacco (35). For example, there is a need for further research to evaluate the long-term effects of smoke-free products on general health. In addition, as complete smoking cessation remains the preferred approach, the ability to effectively identify patients who are able to quit smoking versus those who would benefit from harm reduction, would be useful. Lastly, the number of smoke-free products has increased considerably in recent years, and the contents, quality and relative safety of some of these products are unknown, highlighting the need for further research and appropriate regulation (35).

It is important to note that tobacco companies completely or partially own many of the leading electronic cigarette manufacturers and have invested heavily in the promotion of the tobacco harm reduction approach (36). In light of this, an editorial by Drs Koh and Fiore has suggested three principles that could help overcome disagreements among the various stakeholders and promote progress in this area: (i) the need to devalue cigarettes and other combustibles; (ii) use of approved cessation medications and legally marketed harm reduction products in adults; and (iii) prevention of exposure to tobacco in children, adolescents and young adults (36).

The Expert Panel affirmed the importance of promoting transparent discussion of the scientific evidence regarding harm reduction strategies and cigarette smoking alternatives from both independent and industry sources. However, the Expert Panel emphasized that the cessation of all nicotine- or tobacco-based products remains the ‘gold standard’ towards which healthcare professionals and patients should aim, and underscored the necessity for scientifically substantiated information about smoke-free alternatives for cigarette smokers who wish to continue smoking. This information should derive from Health Authorities, Medical and Scientific Societies, and the Medical Community at large.

### 3.3 Area 3: anti-smoking legislation

The consensus statements regarding anti-smoking legislation highlighted the need for continuing policies that deter people from starting cigarette smoking and encourage them to quit, consistent with WHO guidelines (2). The Expert Panel also agreed that cessation interventions should be proportional to the risks posed by tobacco smoking and smoke-free alternatives, and that an adequate regulatory framework should facilitate the rigorous scientific evaluation of and dissemination of information about novel smoke-free products and their relative health risks.

The Expert Panel did not reach consensus on the statement that a regulatory framework should be developed to offer all smokers the option to transition to smoke-free products, or to allow access to these products to all smokers (with the necessary information provided to allow informed decision-making).

## 4 Discussion

The present Delphi study facilitated rigorous discussion and consensus-building among a panel of experts in various therapeutic areas on the issue of using smoke-free alternatives to cigarettes for tobacco harm reduction in Italy. Profound differences of opinion are known to exist within the medical profession on the question of tobacco harm reduction, as confirmed during this study. This highlights the need for increased efforts to promote dialogue and consensus-building. The Delphi method is a valuable tool for navigating controversies and uncertainties in various scientific fields by providing a structured approach for collecting and synthesizing expert opinions. Importantly, to our knowledge, this is the first Delphi consensus statement to examine the role of smoke-free products in reducing the harm caused by tobacco. The use of the Delphi method in the present study facilitated the delineation of common ground and spotlighted areas of divergence, predominantly those related to tobacco harm reduction. Given the controversial nature of this field, it was not surprising that a clear consensus could not be reached on several statements.

While certain key statements did not achieve consensus in the Delphi process, this disagreement should be viewed as an interpretive opportunity rather than a limitation. Considering the lack of consensus in the broader context of the entire study, it becomes clear that some discrepancies may be due to misinterpretations or incorrect wording of specific questions. This highlights the importance of clear question framing in future studies. At the same time, the existence of disagreements reflects the complexity of the issue and underscores the value of the Delphi method in fostering nuanced discussions and navigating controversies.

The lack of consensus on statement 12 is balanced by the consensus reached in statement 19. This highlights the importance of long-term studies to confirm the reduction of harm with smoke-free alternatives, despite the significant reduction in exposure to toxicants.

The lack of consensus on statement 13 can be attributed to the wording of the question. It appears that some panelists (23%) may have interpreted “exclusive use” as the only option, without any cessation attempts (e.g., NRT use), rather than a complete transition to smoke-free alternatives without dual use with combustible tobacco products. This indicates that clearer formulation of questions is crucial to avoid potential misinterpretations.

The lack of consensus on statement 14 likely stems from an incorrect formulation of the question regarding the FDA statement. The FDA used “modified levels” instead of “decreased levels” of Harmful and Potentially Harmful Constituents (HPHC). This suggests that some panelists highlighted this oversight rather than disagreeing with the FDA’s opinion.

The lack of consensus on statements 35 and 36 is in contrast with the consensus reached for statements 33 and 34. This inconsistency, where the consensus and lack of consensus reached for those statements are in stark contrast to each other, has no explanation other than some panelists could have misinterpreted the relevant questions. It should be noted that the lack of consensus is the result of the peculiar wording used in statements 35 and 36 (where, at any rate, there was an average consensus of 80%) as they referred to “all smokers” and not to “adult smokers who would otherwise continue smoking.” When comparing such a result to the one of statement 21 (100% consensus), the Expert Panel did agree about making smoke-free alternatives accessible to adult smokers who would otherwise continue smoking (with information to enable informed decision-making). However, the take-home message from the cumulative interpretation of such statements is that not all tobacco products have the same risk profile (continuum of the risk concept) and that more restrictive regulations should be applied to products which are potentially more damaging.

The lack of consensus on statements 10 and 11 is apparently in contrast with the consensus reached for statement 20.

On one hand, evidence from recent studies by Qureshi and colleagues (37), Rose and colleagues (38), La Rosa and colleagues (39), and Ansari and colleagues (40) highlights the benefits of smoke-free alternatives in reducing exposure to toxicants and improving health outcomes. On the other hand, findings by several authors underscore unresolved risks, such as the impact of smoke-free products on small airway function (41), tumor metastasis (42), hypertension (43) and cardiorespiratory fitness (44). This duality underscores the contentious nature of tobacco harm reduction and the need for ongoing research and debate to guide evidence-based policies.

However, when interpreted cumulatively, the Panel consensus was clear: both the Harm Reduction approach and assisting adult smokers who would otherwise continue smoking to transition from cigarettes to less damaging options are strategies to be recommended and adopted just in the latter population, whilst cessation remains the gold standard to pursue in all cigarette smokers.

In total, 24 statements (Statements 1, 3–9, 19–21, 23–26, 28–34, 37 and 38) reached consensus after two rounds of voting, indicating substantial agreement on a range of issues within three broad areas (1): harm from tobacco smoking and strategies for harm reduction, (2) smoke-free alternatives to cigarettes and (3) anti-smoking legislation.

Despite reaching a consensus on several statements, the Expert Panel remained divided on several issues. For example, divergent opinions were expressed on statements regarding the role of smoke-free alternatives for harm reduction. These disagreements are reflected in the debate that continues to unfold in the scientific literature (6, 23, 24, 27, 45–47). Proponents for the use of smoke-free alternatives for tobacco harm reduction tend to emphasize that most of the harm associated with tobacco use comes from combustion, which is absent in smoke-free alternatives (48). Others have raised concerns about the safety of the chemicals contained in electronic cigarettes and about the possibility that smoke-free tobacco alternatives may serve as a gateway to traditional cigarettes or cause tobacco smokers who have previously quit to relapse (24, 27, 49). However, the most recent data from the Global Youth Tobacco Survey, which was carried out in Italy over the 2021–2022 school year and questioned 2,069 adolescents aged 13–15 years, has indicated that the use of cigarettes and nicotine products overall has decreased over the past 8 years (50). Further, as other interventions and policies have thus far failed to eliminate smoking, despite substantial public policy efforts, it may be that commitment to the goal of complete abstinence on the part of those who are concerned about smoke-free tobacco alternatives prevents them from seeing the value of intermediate solutions (6).

Ultimately, this Delphi study represents a critical approach to understanding expert perspectives that is consistent with the principles of scientific research. Rather than seeking unanimous agreement, the process illuminated diverse viewpoints and fostered nuanced discussion, which is required to addressing the diverse challenges of tobacco harm reduction.

The concept of abstinence as the only possible strategy in tobacco control needs to be critically reviewed. Undoubtedly, complete cessation of smoking is the best possible outcome. However, this focus necessarily overlooks the needs of those who are unwilling or unable to quit entirely. For these individuals, the use of smoke-free products may provide a suitable, if not the only, strategy to substantially reduce their health risks, and harm reduction measures should be recognized as an essential component of public health strategy.

Hesitancy in adopting harm reduction strategies has also been observed in other areas of public health, such as the opioid crisis, where harm reduction strategies have shown substantial benefits in reducing opioid use, lowering the risk of overdose, and preventing the transmission of infectious diseases. However, despite clear evidence of the benefits of harm reduction measures, many experts and policymakers continue to favor abstinence-only approaches, often sidelining the benefits of non-abstinence strategies (51, 52).

This example provides a clear parallel for tobacco control: failing to recognize the value of harm reduction may limit our ability to effectively reduce the burden of smoking-related diseases.

This study was funded by Philip Morris Italia, a subsidiary of Philip Morris International. The funding reflects a continued commitment by the tobacco industry to develop less harmful alternatives for consumers, indicating a clear paradigm shift toward a harm reduction approach, similar to efforts seen in the automotive industry to improve product safety.

While our study was funded by a tobacco company, several steps were taken to ensure the research’s objectivity and integrity. The independence of the research team, the anonymous voting process, and the involvement of an independent methodologist in data analysis were crucial measures taken to mitigate potential conflicts of interest. Transparency in our methodology and full disclosure of the funding source have been paramount, and we believe these steps maintain high standards of scientific rigor. Additionally, we acknowledge the concern that some potential collaborators might have opted out due to the source of funding, which could have influenced the overall composition of the Scientific Committee. However, the rigorous nature of the DELPHI consensus methodology inherently minimizes the potential impact of any selection bias on our work. Even if significant selection bias were to occur at the Scientific Committee level, the iterative procedural steps of the DELPHI consensus allow for agreements/disagreements from many key opinion leaders. Importantly, the key opinion leaders were selected from a pool of 20 scientific societies, comprising health professionals with a diverse range of views on tobacco harm reduction. This diversity enhances the balance and integrity of our analysis.

These ongoing disagreements reflect the complexities and challenges inherent in forming a consensus on a rapidly evolving and contentious field like tobacco harm reduction. Therefore, the findings of this consensus study underscore the need for further research, dialogue and expert discussions to guide policy and practice in this important public health area. In particular, future work should focus on the statements where consensus was not reached, to examine these areas in more depth, and to provide further evidence to guide policy and practice in tobacco harm reduction in Italy. Indeed, the authors suggest that another consensus study be conducted within the next 2 years, given the large amount of scientific evidence likely to be published in the near future in the field of preventable diseases.

While this research centers around the Italian context, the findings can be applied to any nation facing a significant prevalence of smoking. The concepts of reducing tobacco-related harm and the collaborative consensus-building process discussed are universally applicable, offering a framework that can be tailored to address tobacco use as a public health issue in different countries.

The present consensus study had several limitations. Firstly, consensus was based on the opinions of a select group of 20 specialists, which may not encompass the broader perspectives of the larger scientific community. Secondly, as the study was limited to the opinions of Italian experts, the present consensus may not be readily generalizable to specialists in other countries. However, one of the strengths of the present study was the use of a much higher threshold for consensus (>85%) than is commonly used in Delphi studies.

To ensure the integrity and objectivity of our research, the Expert Panel maintained strict independence from the sponsor working independently throughout all stages of the research process. All the conclusions presented in this study reflect the opinions of the authors and have not been influenced by the sponsor. We have fully disclosed our funding source and are committed to transparency, making our methodology and data available for peer review.

In conclusion, this Delphi consensus study represents an important step in establishing expert consensus on the role of smoke-free products in reducing the harm caused by tobacco smoking in Italy. It provides a framework for ongoing dialogue and action, with the ultimate aim of reducing the significant health burden posed by tobacco smoking.
