# Supplementary material for: Delphi consensus methodology to gauge expert perspectives on smoking prevention, cessation and harm reduction in Italy
Source: Front Psychiatry. 2025 Jan 31;16:1349265. doi: 10.3389/fpsyt.2025.1349265 (PMC11826420; doi:10.3389/fpsyt.2025.1349265)
Supplement: Supplementary file 1 [file DataSheet1.docx]

Supplementary Material

Delphi Consensus Methodology Gauges Expert Perspectives on Smoking Prevention, Cessation and Harm Reduction in Italy

Pasquale Caponnetto*, Vincenzo Contursi, Francesco Fedele, Fabio Lugoboni, Salvatore Novo

*** Correspondence:** P. Caponnetto: [p.caponnetto@unict.it](mailto:p.caponnetto@unict.it)

# Supplementary Data

## Supplementary Methods

### Scientific Committee and Expert Panel members

The Scientific Committee consisted of the following members:

• Prof. Pasquale Caponnetto (Università degli Studi di Catania, Catania, Italy)

• Dr Vincenzo Contursi (ASL Bari, Bari, Italy)

• Prof Francesco Fedele (Istituto Nazionale per le Ricerche Cardiovascolari, Bologna, Italy)

• Dr Fabio Lugoboni (Università di Verona, Verona, Italy)

• Prof Salvatore Novo (AOUP “Paolo Giaccone di Palermo”, Palermo, Italy)

The Expert Panel consisted of the following members (with specialty shown):

• Eugenio Aguglia (Psychiatry; Catania, Italy)

• Leonardo Aluigi (Angiology; Bologna, Italy)

• Pierluigi Antignani (Angiology; Rome, Italy)

• Fabio Bandini (Neurology – Stroke; Genoa, Italy)

• Maria Grazia Cannarozzo (Dentistry; Catania, Italy)

• Gabriele Catena (Cardiology; Teramo, Italy)

• Roberto Crisci (Thoracic Surgery; L’Aquila, Italy)

• Domenico D’Ugo (Oncological Surgery; Rome, Italy)

• Claudio Ferri (Internal Medicine; L’Aquila, Italy)

• Andrea Fontanella (Internal Medicine; Naples, Italy)

• Pietro Lentini (Cardiology; Rome, Italy)

• Dario Manfellotto (Internal Medicine; Rome, Italy)

• Giovanni Martinotti (Psychiatry; Chieti, Italy)

• Gianna Maria Nardi (Dental Hygiene; Rome, Italy)

• Sandro Rengo (Dentistry; Naples, Italy)

### Literature Search Strategy

The PubMed database was searched using the following terms, with no restrictions in terms of publication date or language: ((tobacco[Title/Abstract] OR smok*[Title/Abstract]) OR (Tobacco Products[MeSH Terms] OR Cigarette Smoking[MeSH Terms] OR Smoking[MeSH Terms] OR Tobacco Smoking[MeSH Terms])) AND ((electronic cigarette[Title/Abstract] OR e-cigarette[Title/Abstract] OR vaping[Title/Abstract] OR heated tobacco product[Title/Abstract]) OR (Smoking Devices[MeSH Terms] OR Electronic Nicotine Delivery Systems[MeSH Terms] OR Tobacco, Smokeless[MeSH Terms])) AND ((Harm Reduction[MeSH Terms] OR Tobacco Control[MeSH Terms] OR Smoke-Free Policy[MeSH Terms] OR Smoking Prevention[MeSH Terms] OR Smoking Cessation[MeSH Terms]) OR (harm reduction[Title/Abstract] OR smoking prevention[Title/Abstract] OR smoking cessation[Title/Abstract])) AND ((Italy[Title/Abstract] OR Italian[Title/Abstract]) OR (Italy[MeSH Terms] OR Italian people[MeSH Terms]))

# Supplementary Figures


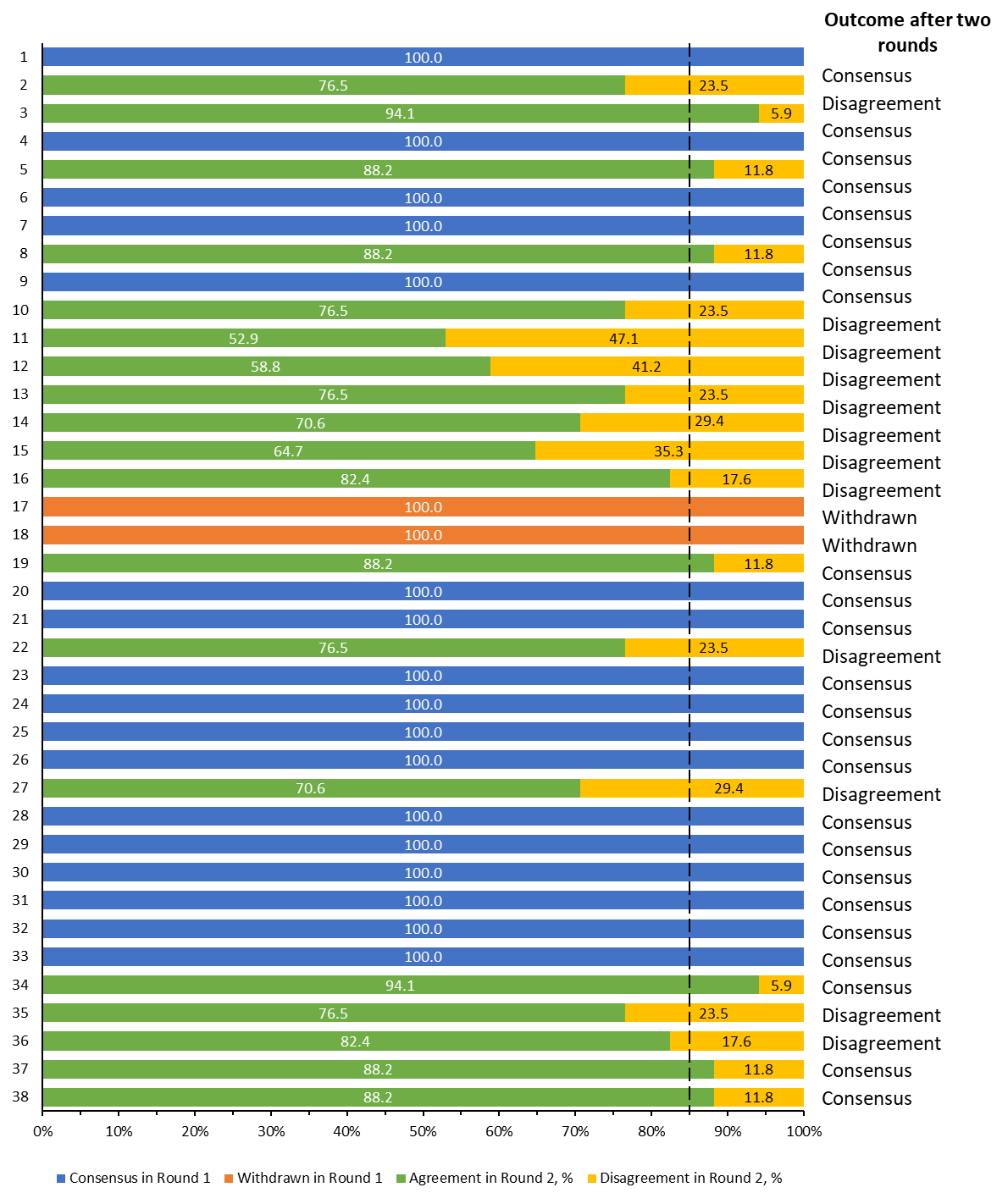


**Supplementary Figure 1.** Voting outcomes after two rounds. Agreement indicates the percentage of respondents whose ratings fell into the same agreement category, i.e. low agreement (Likert scale scores 1–3), moderate agreement (scores 4–6), high agreement (scores 7–9).

Disagreement indicates the percentage of respondents who expressed high or low agreement plus the percentage of respondents who expressed moderate agreement.

The dashed line indicates the consensus threshold of 85%.
